# Potential impacts of landuse changes on the supply–demand relationship of water resources in semiarid loess hilly regions

**DOI:** 10.1038/s41598-026-40006-7

**Published:** 2026-02-26

**Authors:** Longtan Qiao, Qiang Li, Haoxuan Zhang, Zixuan Zhang, Hanwen Yang, Yilong Zhu, Zhengzheng Mao

**Affiliations:** 1 Xi’an Center of Mineral Resources Survey, China Geological Survey, Xi’an, 710100 China; 2 Qinling Loess Plateau Transition Zone Observation and Research Station for Coupling of Soil and Water Elements and Conservation of Biological Resources, Xi’an, 714000 China; 3 Xi’an Institute of Geological and Mineral Exploration Co., Ltd, 710100 Xi’an, China

**Keywords:** Landuse change, Mitigation strategies, Multi-scenario simulation, PLUS-InVEST model, Supply–demand risks, Agroecology, Environmental impact

## Abstract

In ecologically fragile semiarid loess hilly agricultural regions, water resources constitute a critical constraint on sustainable development. Previous studies have demonstrated that landuse changes significantly affect the spatiotemporal distribution of water through vegetation cover modifications and hydrological process shifts. This study aims to predict future landuse changes and assess their impacts on water supply and demand, thereby providing a basis for sustainable water resource management. The current study employed an integrated PLUS-Markov chain approach (with a high validation accuracy, OA > 0.9 and Kappa > 0.83) complemented by the InVEST model to project landuse arrangements under three scenarios (NIS, FSS, and EDS) for Guyuan city in 2030, 2040, and 2050, and to analyze the consequent spatiotemporal evolution of water supply and demand risks. The results indicated that by 2050, the cropland under the NIS scenario decreased by 6.7%, primarily transitioning to grassland. In contrast, the FSS scenario led to a substantial increase in cropland by 10.7%, resulting in an overall reduction in built-up area. Meanwhile, the EDS scenario drove rapid urbanization, with a built-up area expansion rate reaching 2.99 km²/year, largely at the expense of cropland. By 2050, landuse change was projected to exert minor influences on the regional water supply, with only a 7.8% variation projected compared with 2030 levels, whereas substantial impacts were projected for the water demand, which increased by 43.3% during the same period. Notably, approximately 90% of Guyuan’s area may face water security risks by 2050, particularly in ecological reserves and urban zones, with the risk severity increasing over time. Several adaptive strategies were proposed to reconcile land–water relationships, thereby offering practical solutions for sustainable agroecosystem management in semiarid loess hilly regions.

## Introduction

Water, a strategic resource and fundamental environmental element^1^, supports human survival and ecological security through sustainable use. Population growth and economic development exacerbate water scarcity and aquatic ecosystem degradation, significantly constraining agricultural production and sustainable water use^2^. In semiarid regions with intense competition among ecological, agricultural, and domestic water use^3,4^, climate change amplifies drought risks through enhanced evapotranspiration and precipitation variability^5^, which destabilizes agricultural systems by widening supply–demand gaps. Crops in semiarid loess hilly regions exhibit heightened water stress sensitivity, with fragile ecosystems exhibiting low resilience, particularly in areas with rapid landuse changes^6,7^. Systematic assessments of water supply–demand dynamics are essential for reconciling agricultural development with ecological preservation in these regions. Recent research has focused mainly on spatiotemporal pattern analysis in localized areas^8–10^but lacks comprehensive evaluations of supply–demand dynamics, future scenario projections, and a mechanistic understanding of risk evolution.

As a pivotal driver of environmental change, landuse change profoundly influences regional resource allocation patterns through its evolving spatial and temporal dynamics^11,12^. Hydrologically, landuse change affects watershed-scale water fluxes by altering surface cover characteristics and regulating evapotranspiration rates, soil infiltration efficiency, and surface retention capacity, thereby causing dynamic fluctuations in water yields^6^. Socioeconomically, landuse change causes the redistribution of the water demand across functional zones by restructuring three life spaces (production, ecological, and living spaces), altering their spatial demand characteristics^13^. Researchers have developed modeling systems (CA, FLUS, and CLUE) to simulate landuse changes in complex geographical environments. The PLUS model integrates patch-generation mechanisms and multi-agent decision systems within a spatially explicit framework, demonstrating unique capabilities in simulating heterogeneous landscape evolution, particularly for multi-scale landuse change scenarios^14^.

Under global climate change, the northward shift in agricultural production patterns and the marginal expansion of cultivated land generate multidimensional stress effects, exacerbating spatial competition among water–food–energy systems^15,16^. Guyuan city, a representative semiarid loess hilly area in China with fragile ecosystems, faces water scarcity that constrains economic resource allocation, which restricts sustainable agricultural intensification and poses a threat to ecological barrier stability. In this study, our objectives are to (1) simulate landuse patterns in Guyuan from 2030 to 2050 under three scenarios via the PLUS model, namely, a natural increase scenario (NIS), a food security scenario (FSS), and an economic development scenario (EDS); (2) quantify water supply–demand dynamics and assess spatiotemporal variations in future water security risks; and (3) investigate landuse-driven mechanisms of water risk and propose adaptive agricultural strategies and ecological management approaches for loess semiarid regions.

## Analytical methods

### Study area

**Fig. 1:**
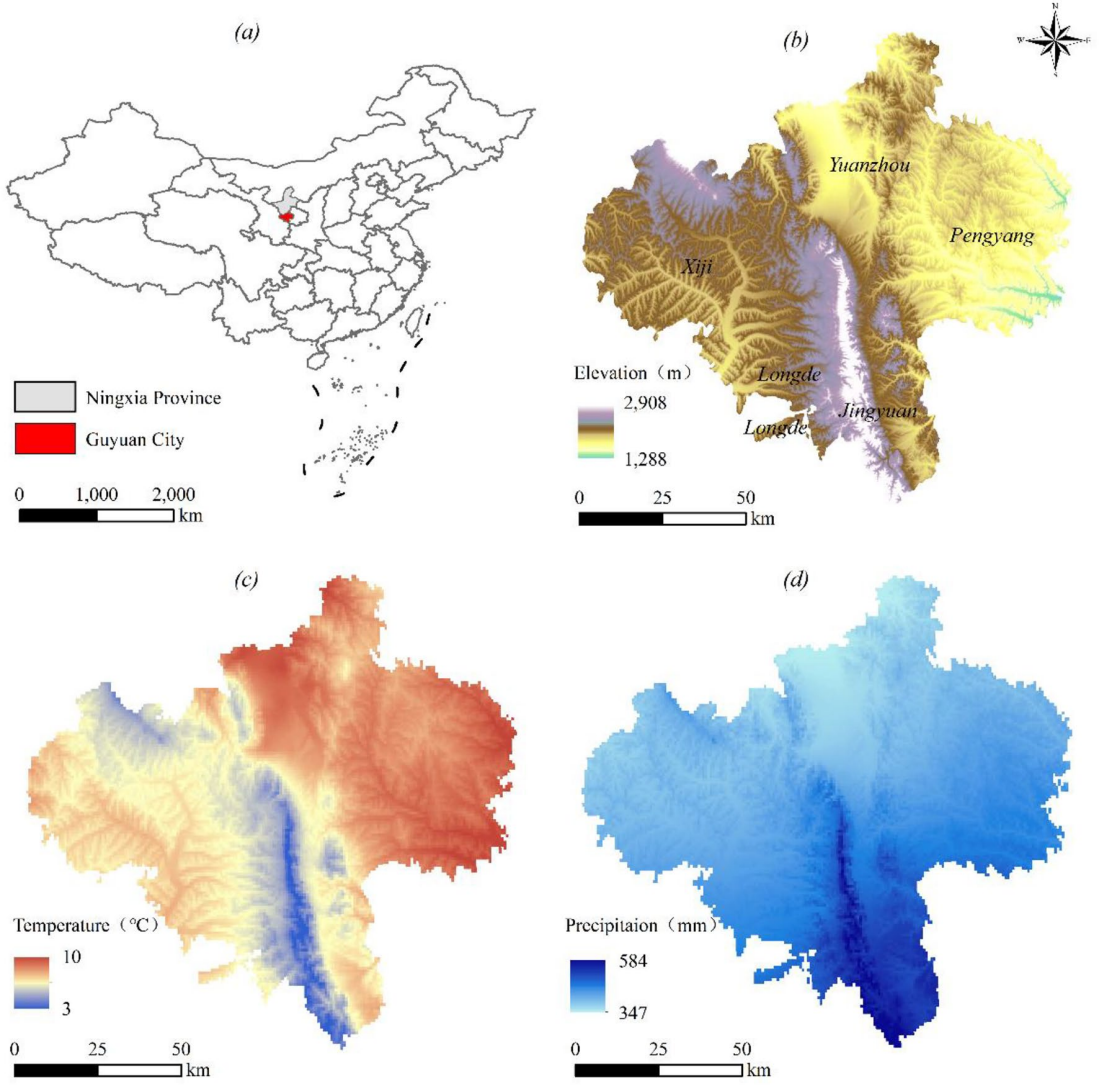
Key geographical and climatic features of Guyuan city: (**a**) Regional location, (**b**) DEM and county distribution, (**c**) mean annual temperature, and (**d**) mean annual precipitation. Created with ArcGIS 10.8.

Guyuan city (105°19′–106°57′E, 35°14′–36°31′N) occurs in the semiarid loess hilly region of southern Ningxia, China. Covering 10,500 km², it includes five counties, namely, Xiji, Longde, Jingyuan, Pengyang, and Yuanzhou. Situated at the edge of China’s Loess Plateau, Guyuan’s terrain is divided by the Liupan Mountains into eastern and western sections, featuring high topography in the south and low topography in the north with complex gullies and hills instead of plains, which is characteristic of arid loess landscapes. With a warm-temperate semiarid continental climate, this region experiences annual temperatures of 3–10 °C and receives 300–600 mm of precipitation. Details are shown in Fig. 1. The total water resources reach 5.97 × 10^8^ m³, with surface water constituting 5.56 × 10^8^ m³. Agricultural irrigation (1.01 × 10^8^ m³), industrial use (0.06 × 10^8^ m³), and domestic consumption (0.19 × 10^8^ m³) account for 78.12%, 4.83%, and 14.88%, respectively, of the total usage^17^. Guyuan contains the only mountain–forest–steppe ecosystem in northwest China, where the Liupan Mountains serve as a critical barrier against southern expansion of the Tengger Desert.

### Data acquisition and processing

**Fig. 2:**
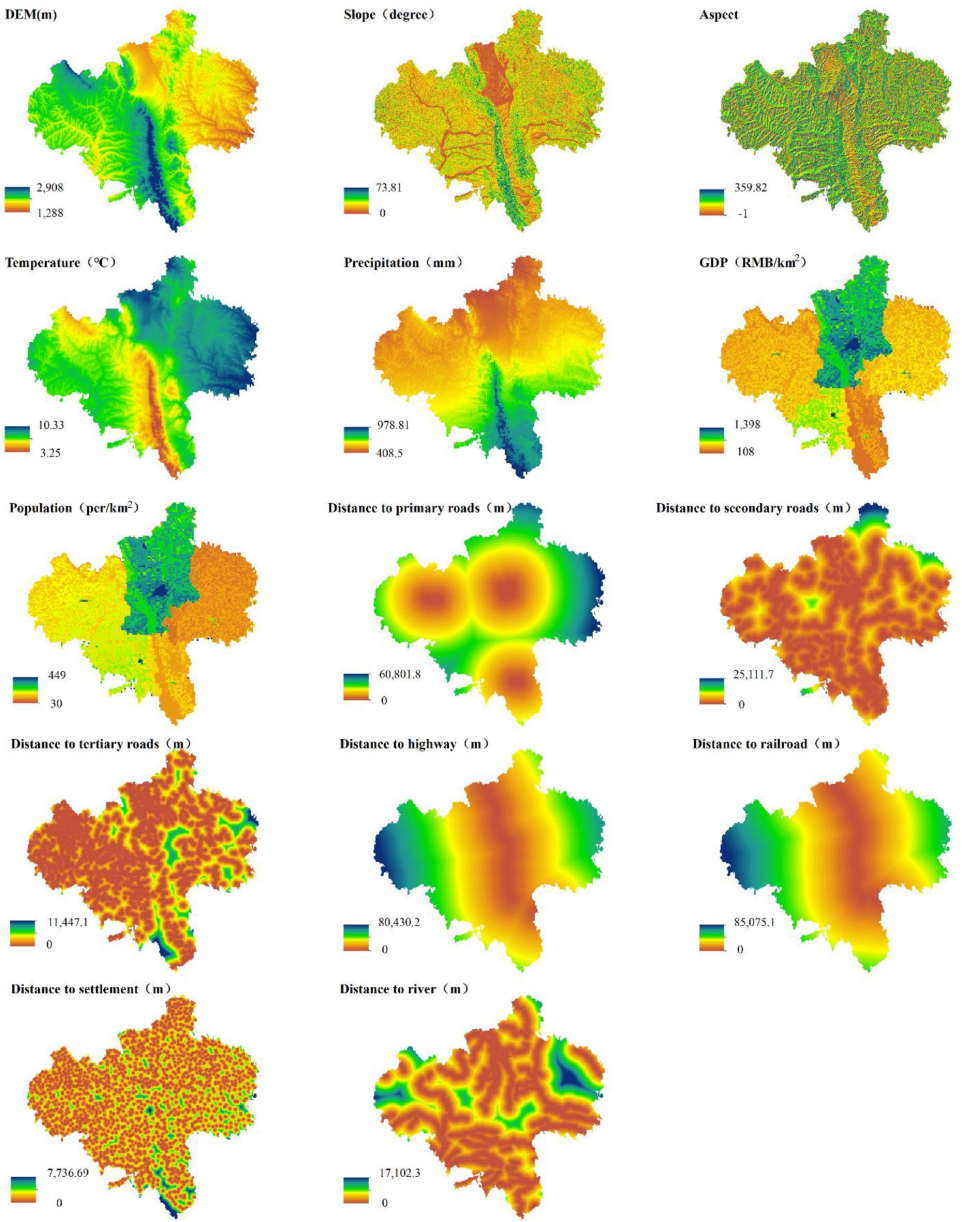
Spatial distribution of key landuse drivers. Created with ArcGIS 10.8.

Landuse data for 2000, 2010, and 2020 at a 30-m spatial resolution were obtained from the Resource and Environment Science Data Center (RESDC) of the Chinese Academy of Sciences^18^. The dataset features a two-tier classification system: 6 primary landuse categories are subdivided into 25 secondary types, with the overall accuracy exceeding 90%^19,20^. Fourteen drivers of landuse change (5 natural geographic factors, 2 socioeconomic factors, and 7 accessibility factors) were selected at a resolution of 30 m (Table 1; Fig. 2). Natural geographic factors included elevation, slope, aspect, mean annual temperature, and precipitation. Socioeconomic factors included the gross domestic product (GDP) and population density, and accessibility factors included vector proximity to primary roads, secondary roads, tertiary roads, railways, highways, rivers, and settlements. Elevation data (30-m DEM) were obtained from the Geospatial Data Cloud^21^, with slope and aspect derived using ArcGIS 10.8. Temperature and precipitation data (1-km grids) were sourced from the Earth Resource Data Cloud^22^. Socioeconomic datasets (1-km grids for the GDP and population density data) were acquired from RESDC^23^. Accessibility vectors (1-km resolution) from the National Geographic Resources Directory were converted into Euclidean distance raster data in ArcGIS10.8^24^. All datasets were resampled to a 30-m resolution and reprojected to WGS_1984_UTM_Zone_48N for spatial consistency.

**Table 1:** Driving factors of landuse Spatial patterns.

Data type	Driving factor	Year	Spatial resolution
Natural geographic	Altitude	2010	30 m
	Elevation	2010	30 m
	Slope direction	2010	30 m
	Average annual temperature	2000–2020	1 km
	Average annual rainfall	2000–2020	1 km
Socioeconomic	GDP	2020	1 km
	Population density	2020	1 km
Accessibility	Distance to primary roads Distance to secondary roads Distance to tertiary roads Distance to highways Distance to railroads Distance to residential areas Distance to rivers	2020 2020 2020 2020 2020 2020 2020	30 m

Assessing the water supply–demand data of Guyuan required several datasets (Table 2) and multiple calculation variables. Soil properties and depth data (1-km resolution, 2009) were sourced from the Global Soil Database^25^. Monthly potential evapotranspiration data (2000–2020) were obtained from China’s National Earth System Science Data Center^26^. Water use indicators (2000–2020), including per capita domestic consumption, water consumption per 10,000 yuan of the GDP, and irrigation use per hectare, were extracted from the Ningxia Water Resources Bulletin^17^. Annual population and GDP statistics (2000–2020) were acquired from the Ningxia Statistical Yearbook^27^.

**Table 2:** Data required for calculation of the water supply–demand.

Variables	Year	Data sources
Soil properties	2009	Harmonized World Soil Database
Soil depth	2009	Harmonized World Soil Database
Monthly potential evapotranspiration	2000–2020	National Earth System Science Data Center
Monthly precipitation	2000–2020	National Earth System Science Data Center
Available soil moisture	2000–2020	Calculated from Harmonized World Soil Database
Per capita water consumption	2000–2020	Ningxia Water resources Bulletin
Water consumption per 10,000 yuan of the GDP	2000–2020	Ningxia Water resources Bulletin
Irrigation water use per acre of cultivated land	2000–2020	Ningxia Water resources Bulletin
Permanent resident population	2000–2020	Ningxia Statistical Yearbook
GDP data of Guyuan city	2000–2020	Ningxia Statistical Yearbook

### Modeling and analytical methods

**Fig. 3:**
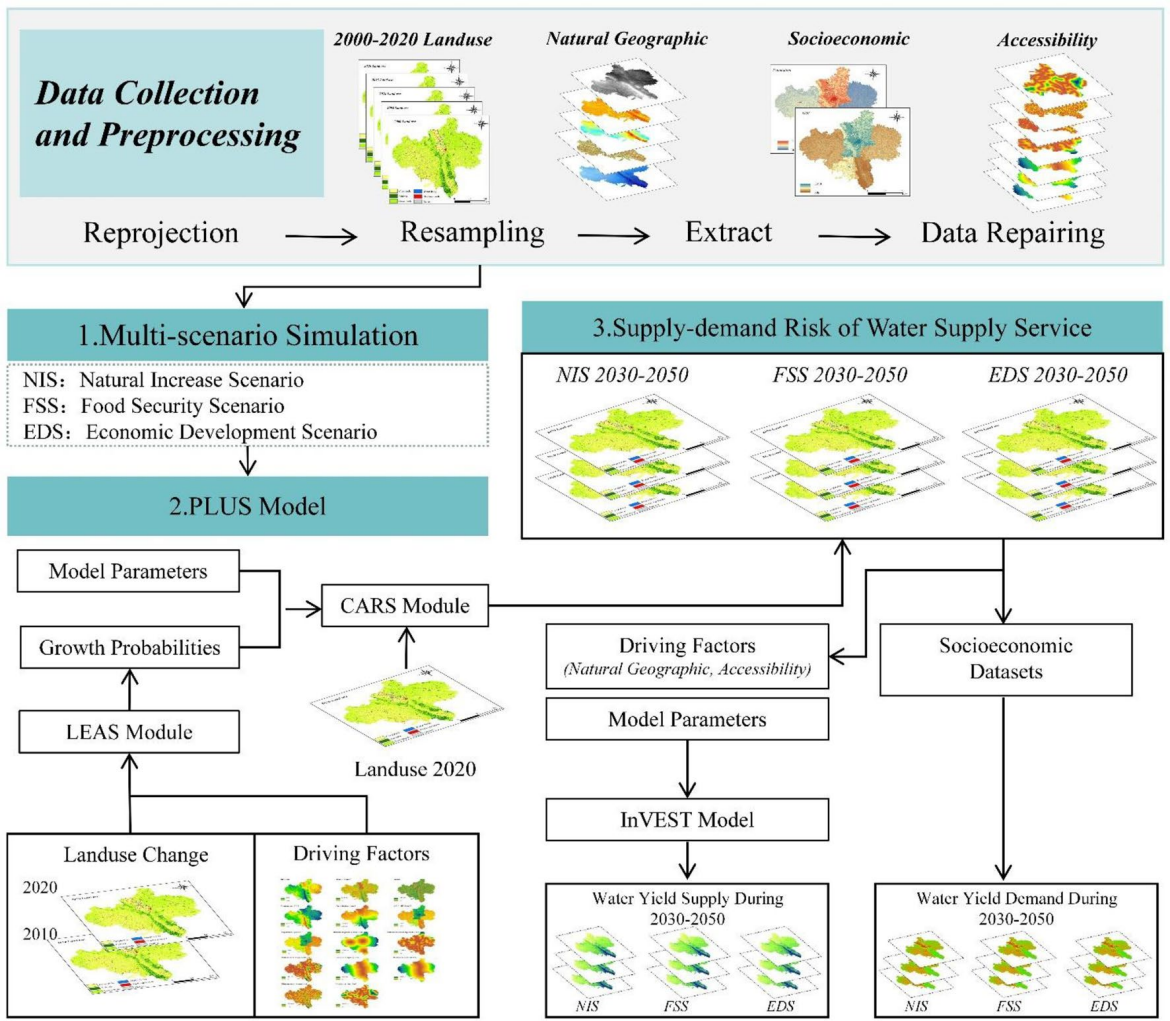
Technical workflow for landuse modeling and water yield assessment. The maps were created with ArcGIS 10.8.

In this study, we analyzed landuse patterns in Guyuan under multiple scenarios and evaluated water supply–demand dynamics to provide guidance for sustainable urban agriculture. The framework is shown in Fig. 3:

#### PLUS model and scenario design

The PLUS model, developed by Liang et al.^28^, is an extension of the FLUS model^29^and integrates raster data processing and cellular automata to simulate landuse changes on a patch-level resolution. The model features two core components: the Land Expansion Analysis Strategy (LEAS) module and the Cellular Automata with Random Seeds (CARS) module. These modules enable high-precision landscape pattern simulations while revealing underlying landuse change mechanisms. Via the use of 2010–2020 landuse patterns as baseline data, we simulated 2030–2050 spatial distributions in Guyuan under multiple scenarios.

(1) LEAS module.

The LEAS module aims to identify landuse patterns through the temporal analysis of sequential landuse data. It aims to extract change areas by category, training data are sampled randomly, and random forest classification (RFC) is applied to quantify expansion probabilities and driver impacts. Key parameters were optimized for accuracy and efficiency^30^: bootstrap sampling (0.2), decision trees (60), and max features (4, based on driver quantity)^31^. The LEAS framework enhances the operational efficiency by simplifying complex processes, enabling systematic analysis of landuse change drivers and supporting robust scenario simulations.

(2) CARS module.

The CARS module integrates stochastic patch-generation mechanisms for multi-landuse simulations^32,33^. The simulation relies on adaptive inertia mechanisms to regulate local landuse competition, ensuring convergence to projected future demands. The CARS module enhances conventional CA through stochastic seed generation and threshold decay mechanisms, enabling multi-category landuse evolution modeling. When neighborhood effects reach zero, the mechanism provides development probabilities across land types as seed values. The PLUS model then auto-generates simulated patches constrained by these probabilities. Key inputs include Patch generation threshold, Expansion coefficient, Transition matrix and Neighborhood weights.

The Patch generation threshold is an indicator reflecting the difficulty of landuse conversion, with values ranging from 0 (indicating easy conversion) to 1 (indicating difficult conversion). The Expansion coefficient represents the tendency for new patches to emerge, with values ranging from 0 (indicating easy generation) to 1 (indicating difficult generation). In the simulation spanning from 2000 to 2030, the Patch generation threshold and Expansion coefficient are set to 0.8 and 0.1, respectively. Beginning in 2030, the Patch generation threshold decreases by 0.1, while the Expansion coefficient increases by 0.1 every ten years, in order to accommodate future landuse demands.

The Transition matrix reflects whether a specific landuse type can be transformed into other types, where a value of 0 denotes prohibition and 1 denotes permission. Based on the analysis of landuse dynamics in Guyuan City between 2000 and 2020, three transfer matrices have been established for the specified scenarios, as detailed in Table 3.

**Table 3:** Multi-scenario transition matrix.

Land Type	Scenarios	Croplands	Forests	Grasslands	Water	Built-up land	Barren
Croplands	NIS	1	1	1	1	1	1
	FSS	1	1	1	1	1	1
	EDS	1	1	1	1	1	1
Forests	NIS	1	1	1	1	1	1
	FSS	1	1	1	1	1	1
	EDS	1	1	1	1	1	1
Grasslands	NIS	1	1	1	1	1	1
	FSS	1	1	1	1	1	1
	EDS	1	1	1	1	1	1
Water	NIS	0	0	0	1	0	0
	FSS	1	0	0	1	0	0
	EDS	0	0	0	1	1	0
Built-up land	NIS	0	0	0	0	1	0
	FSS	1	0	0	0	1	0
	EDS	1	1	1	1	1	1
Barren	NIS	1	1	1	1	1	1
	FSS	1	1	1	1	1	1
	EDS	1	1	1	1	1	1

Neighborhood weights (0–1 scale) reflect the radii of land-type influence zones. Under the NIS, weights were derived from historical conversion ratios (2000–2020) between original and changed areas^30^. Under the FSS, the cropland transition rates to non-cropland types were amplified to 1.2 the baseline values (domain weight = 1). Forest land neighborhood weights were subjected to a 46% upscaling, whereas grassland domain weights were attenuated by 58%; the adjustments were calibrated to historical cropland transition patterns (2000–2020). Conversely, the EDS aimed to suppress built-up land conversion rates to 0.85× baseline levels, decreasing the weights of other land categories at variable rates. The scenario-specific neighborhood weight configurations are provided in Table 4.

**Table 4:** Scenario-specific neighborhood weighting configurations.

Scenarios	Croplands	Forests	Grasslands	Water	Built-up land	Barren
NIS	1	0.28	0.41	0.03	0.29	0.01
FSS	1	0.41	0.17	0.04	0.38	0.01
EDS	1	0.29	0.36	0.03	0.32	0.01

#### Multi-scenario simulation analysis

Three scenarios were modeled, i.e., a natural increase scenario (NIS), a food security scenario (FSS), and an economic development scenario (EDS), as specified in Table 5.

Scenario 1, i.e., NIS, serves as a natural development scenario. For the 2030 landuse simulation projections, transfers between land types were based on the 2010–2020 extents of land expansion and drivers and were used as a basis for the other scenario settings.

Scenario 2, i.e., FSS, focuses on advancing farmland protection and food security. In the simulation process, the chance of the conversion of farmland to other landuse is reduced by 20%, and the chance of the conversion of developed land, watersheds, grasslands, forests, and unused land into farmland is increased by 10%.

Scenario 3, i.e., EDS, focuses on satisfying economic development needs to increase the urbanized area. During the simulation, the chance of the conversion of developed land to other landuse is reduced by 15%, and the chance of other land types shifting to developed land is increased by 10%.

**Table 5:** Probability of landuse conversion in future scenarios.

Scenario	Landuse Change	Conversion Probability Adjustment
NIS	Landuse conversion probabilities remain unchanged from 2000–2020	Baseline transition probability
FSS	Croplands→Forests	−20%
	Croplands→Grasslands	−20%
	Croplands→Water body	−20%
	Croplands→Built-up land	−20%
	Croplands→Barren land	−20%
	Forests→Croplands	+ 10%
	Grasslands→Croplands	+ 10%
	Water body→Croplands	+ 10%
	Built-up land→Croplands	+ 10%
	Barren→Croplands	+ 10%
EDS	Built-up land→Croplands	−15%
	Built-up land→Forests	−15%
	Built-up land→Grasslands	−15%
	Built-up land→Water body	−15%
	Built-up land→Barren land	−15%
	Croplands→Built-up land	+ 10%
	Forests→Built-up land	+ 10%
	Grasslands→Built-up land	+ 10%
	Water body→Built-up land	+ 10%
	Barren→Built-up land	+ 10%

#### Water supply and demand calculations

(1) Water supply.

**Fig. 4:**
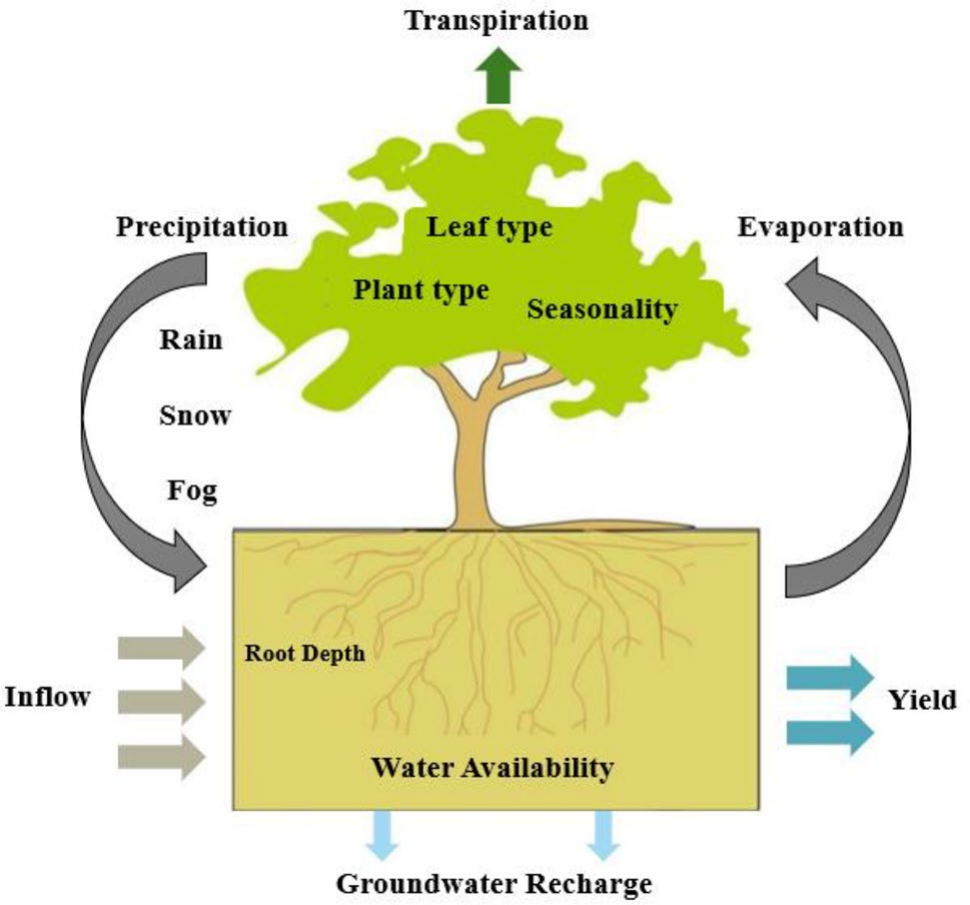
Conceptual diagram of the InVEST water yield model.

As a critical ecosystem service, the water yield is integral to ecohydrological systems. It refers to the replenishable water supply generated by ecosystems for human utilization^34,35^. The raster data of landuse under multiple scenarios from 2030 to 2050, generated by the PLUS model, are utilized as input parameters for the InVEST water yield module. Additionally, preprocessed data including annual average precipitation, annual average evapotranspiration, root restriction layer depth, plant available water content, and regional boundary vector data are imported. Model parameters are configured to calculate the water yield at the raster level for each scenario. The InVEST water yield model primarily estimates water yield based on the Budyko thermohydrological coupling balance hypothesis (Fig. 4). The annual water yield is calculated as the difference between total annual precipitation and total annual evapotranspiration. The model operates on the basis of landuse raster data to determine the annual water yield for each raster unit within the study area. The specific calculation formula is as follows^14^:

1 $${Y_x} = {P_{_X}} \times \left( {1 - \frac{{AE{T_x}}}{{{P_x}}}} \right)$$

23where *Y*_*x*_ is the annual water yield (mm), *AET*_*x*_ is the actual evapotranspiration (mm), and *P*_*x*_ is the precipitation (mm) for grid cell *x*.

where *R*_*x*_ denotes the Budyko aridity index (potential evapotranspiration/precipitation ratio) for a given grid cell, and ω_x_ denotes a dimensionless parameter that integrates climate and soil properties. Moreover, *AWC*_*x*_ denotes the plant-available volumetric soil moisture, and *Z* is the empirically determined Zhang coefficient (1 ≤ *Z* ≤ 20)^14^.

4The soil parameters are as follows: *SAN%* is the sand fraction, *SIL%* is the silt fraction, *CLA%* is the clay fraction, and *C%* is the soil organic carbon concentration. Croplands and grasslands exhibit better water supply capacities than woodlands do, and both landuse types commonly exhibit lower evapotranspiration and shallower root systems.

(2) Water demand.

Guyuan city’s water demand encompasses three primary sectors, i.e., domestic daily use, economic development needs, and irrigation for agriculture, which can be expressed as follows^28^:

56

7 $$po{p_{xy}} \times {d_x} + gd{p_{xy}} \times {e_x} + ag{r_{xy}} \times {f_x}$$

where *p*_*xy*_ and *g*_*xy*_ are the original population and GDP of raster cell *y* in year *x*, respectively; *POP*_*x*_ and *GDP*_*x*_ are the annual regional aggregates for the corresponding years from the Ningxia Statistical Yearbook; *pop*_*xy*_ and *gdp*_*xy*_ are the calibrated population and GDP data of raster cell *y* in year *x*, respectively; and *agr*_*xy*_ is the cultivated land area of raster cell *y* in year *x*. Moreover, *d*_*x*_ and *e*_*x*_ and *f*_*x*_ are the per capita household water use, water use per 10,000 yuan of the GDP, and water use per acre of irrigated farmland in year *x*, respectively. Via the use of historical total population and GDP records from 2000 to 2020, projections for 2030, 2040, and 2050 were derived via linear regression. The PLUS model was used to simulate the future cropland distribution under various scenarios for these target years. Model calibration involved incorporating the projected population and GDP data specific to each respective year. In this study, we calculated the water demand in Guyuan city, focusing on residential and production sectors. Areas proximate to industrial and residential zones typically exhibited high water demand due to concentrated usage. Conversely, unused land, characterized by minimal domestic and production activity levels, demonstrated lower associated water requirements.

#### Water security risk appraisal

Maron et al. (2017) established a quantitative evaluation system for ecosystem service threats^36^and proposed a regional risk analysis framework^34,37^. This framework can be used to systematically assess water risks using four dynamic indicators: supply–demand ratio, ratio evolution trend, supply dynamics, and demand dynamics. The supply–demand ratio serves as the core parameter, revealing spatial variations in water resource conflicts. The calculation is as follows^36^:

8where *R*_*i*_ is the water supply-water demand equilibrium degree of raster cell *i*, and *WY*_*i*_ and *WD*_*i*_ are the supply and consumption of water ecological services, respectively, of cell *i*. *R*_*i*_ *= 0* indicates that the cell is no longer providing water-producing services; 0 < *R*_*i*_ < 1 indicates that the water supply of the cell exhibits a state of deficit, and *R*_*i*_ ≥ 1 indicates that the water supply of the cell meets the demand.

The ratio evolution trend reflects relative supply–demand changes and is as follows:

9where *R*_*tr*_ denotes the direction and intensity of the evolution of the water resource system of the time period between *t*_*1*_ and *t*_*2*_, and *R*_*t1*_ and *R*_*t2*_ denote the ecological service supply and demand balance coefficients in the years *t*_*1*_ and *t*_*2*_, respectively. For *R*_*tr*_ < 0, the unit supply–demand balance coefficient shows a significant decreasing trend; for *R*_*tr*_ ≥ 0, the water resources system maintains stability or shows sustainable improvement. In addition, water supply dynamics and water demand dynamics represent the net changes in water resources^38^, which can be expressed as follows:

1011where *S*_*tr*_ and *D*_*tr*_ are the water supply service and water demand service differentials, respectively; *WY*_*t1*_ and *WY*_*t2*_ are the water supply services in years *t*_*1*_ and *t*_*2*_, respectively; and *WD*_*t1*_ and *WD*_*t2*_ are the water demands in years *t*_*1*_ and *t*_*2*_, respectively. The demand for water decreased when *S*_*tr*_ and *D*_*tr*_ were greater than 0 and increased or remained unchanged when *S*_*tr*_ and *D*_*tr*_ were less than 0. Water supply–demand risk was stratified into seven levels^39^(Table 6) through the analysis of three metrics: (1) the supply–demand ratio, (2) trend of supply–demand ratio, (3) supply–demand dynamics.

**Table 6:** Water ecosystem service supply–demand risk grading.

Grade code	Risk grade	Water supply–demand ratio	Trend in the water supply–demand ratio	Supply–demand dynamics
I	Extinct/Dormant	*R*_*i*_ = 0	— —	— —
II	Critically endangered	0<*R*_*i*_<1	*R*_*tr*_<0	*S*_*tr*_<0, *D*_*tr*_≥0
III	Endangered	0<*R*_*i*_<1	*R*_*tr*_<0	*S*_*tr*_<0, *D*_*tr*_<0 or *S*_*tr*_≥0, *D*_*tr*_≥0
IV	Dangerous	0<*R*_*i*_<1	*R*_*tr*_≥0	*D*_*tr*_<0, *S*_*tr*_<0 or *S*_*tr*_≥0, *D*_*tr*_≥0
V	Undersupplied	0<*R*_*i*_<1	*R*_*tr*_≥0	*S*_*tr*_≥0, *D*_*tr*_<0
VI	Vulnerable	*R*_*i*_≥1	*R*_*tr*_<0	— —
VII	Safe	*R*_*i*_≥1	*R*_*tr*_≥0	— —

## Results

### Verifying simulation accuracy

**Fig. 5:**
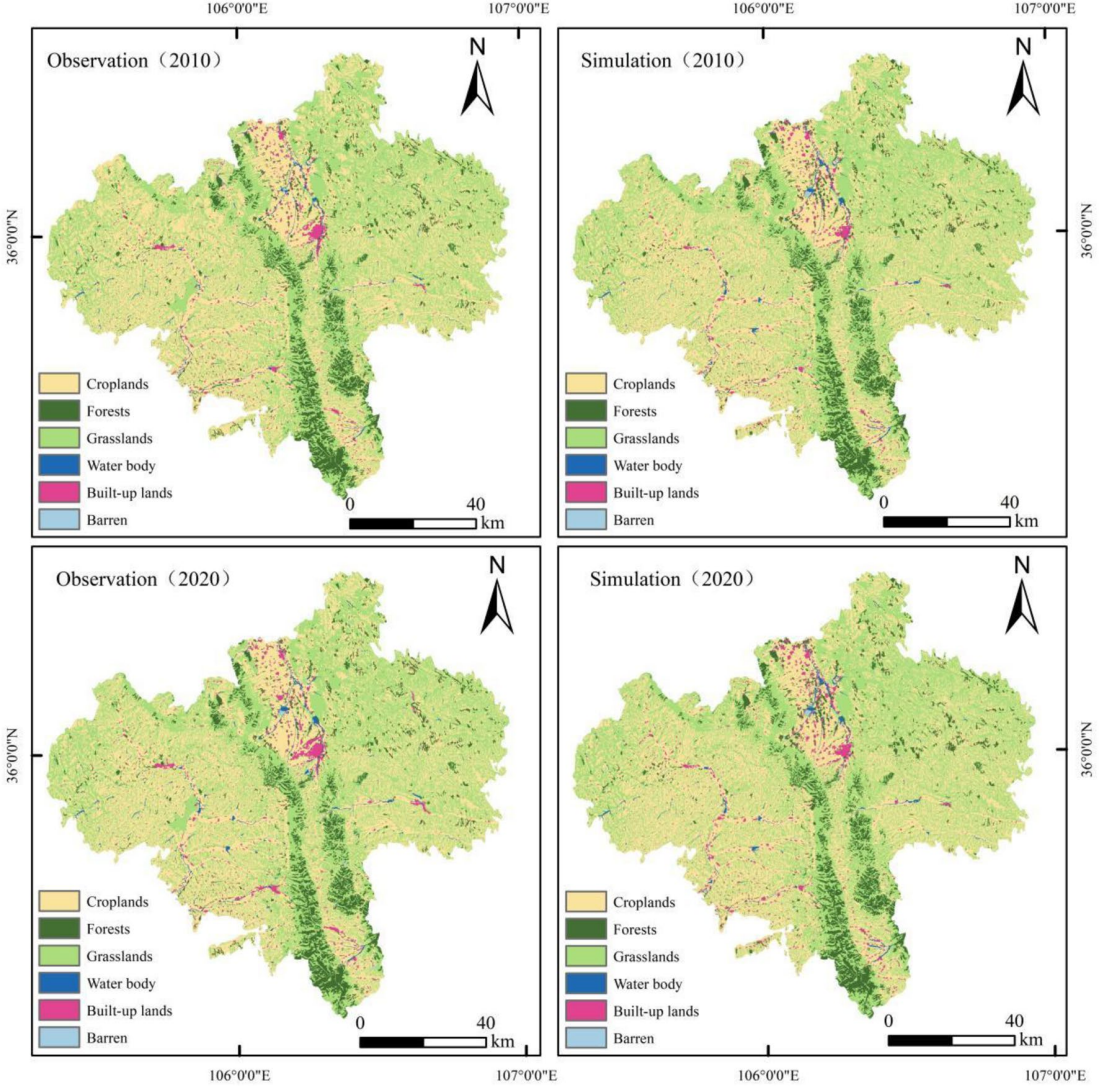
Spatial congruence validation of observed vs. PLUS-simulated landscapes (2010–2020).Created with ArcGIS 10.8.

The simulated landuse patterns closely matched the observations. Via the use of 2000–2005 data as input, the PLUS model was used to generate landuse maps for 2010–2020 (Fig. 5). Built-up areas and water bodies exhibited the highest accuracy, while the patterns of croplands, forests, grasslands, and unused lands maintained high spatial consistency with actual distributions. Quantitative validation using overall accuracy (OA), kappa, and figure of merit (FoM) metrics (Table 7) confirmed the reliability of the model for multi-scenario predictions. From 2010 to 2020, OA and kappa first decreased then increased, reaching 0.914 and 0.856, respectively, by 2020. The FoM metric (0.103 in 2010) reflects pixel-level consistency between the simulated and observed maps. This result agrees with the findings of Pontius, R. G^40^, who argued that FoM values are positively correlated with the net rate of change, which was simulated as 13.59% for the 2000–2020 period in this study; the study period is not long compared with those in studies with higher FoM values^41^. The FoM value of 0.103 does not significantly differ from that in other simulation studies^42,43^.

**Table 7:** Accuracy metrics for the 2010–2020 simulations.

Year	OA	Kappa	FoM
2010	0.912	0.851	0.103
2015	0.902	0.834	0.122
2020	0.914	0.856	0.117

### Multiscenario simulation analysis

**Fig. 6:**
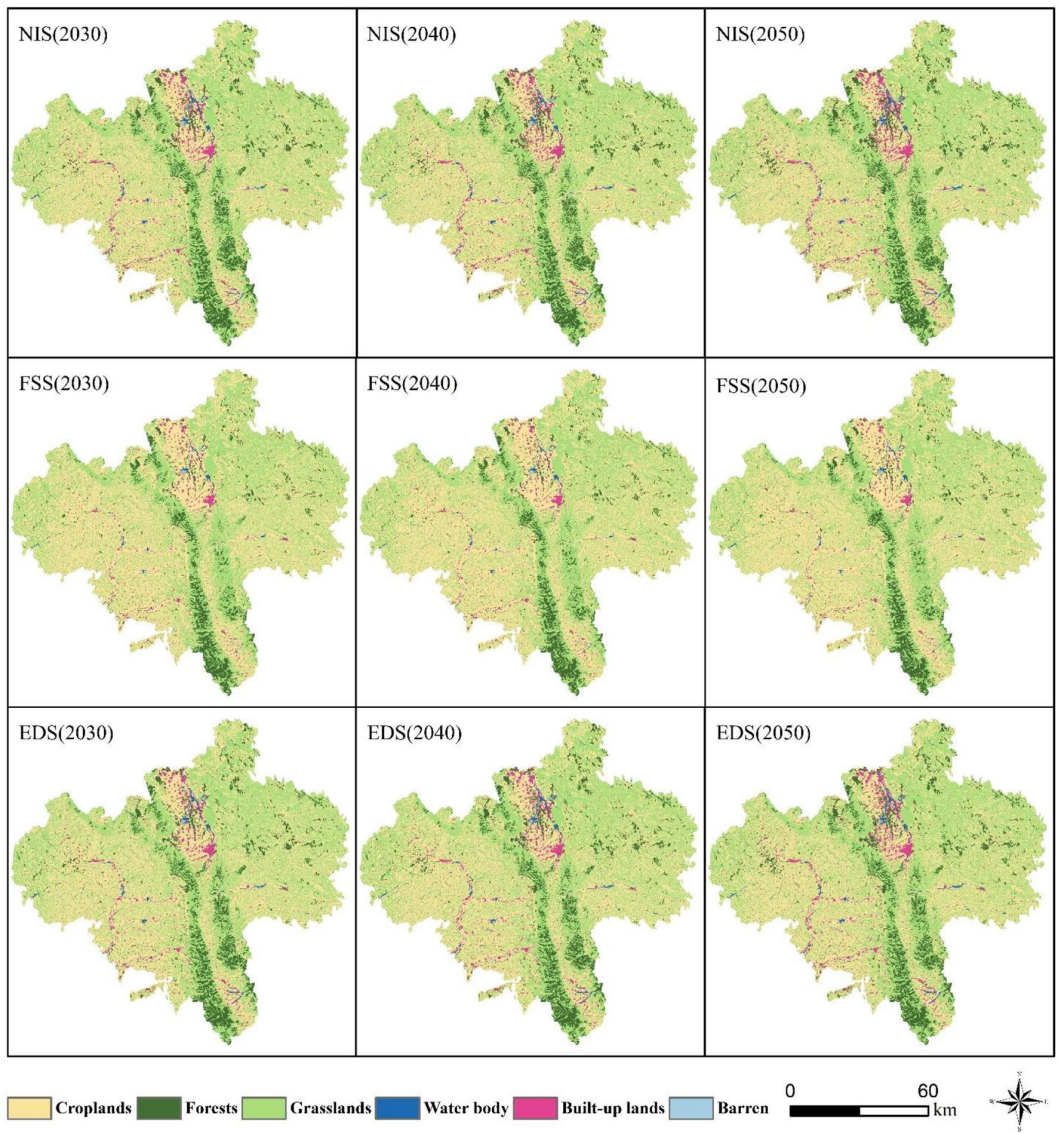
Multi-scenario landuse patterns in Guyuan (2030–2050). Created with ArcGIS 10.8.

**Fig. 7:**
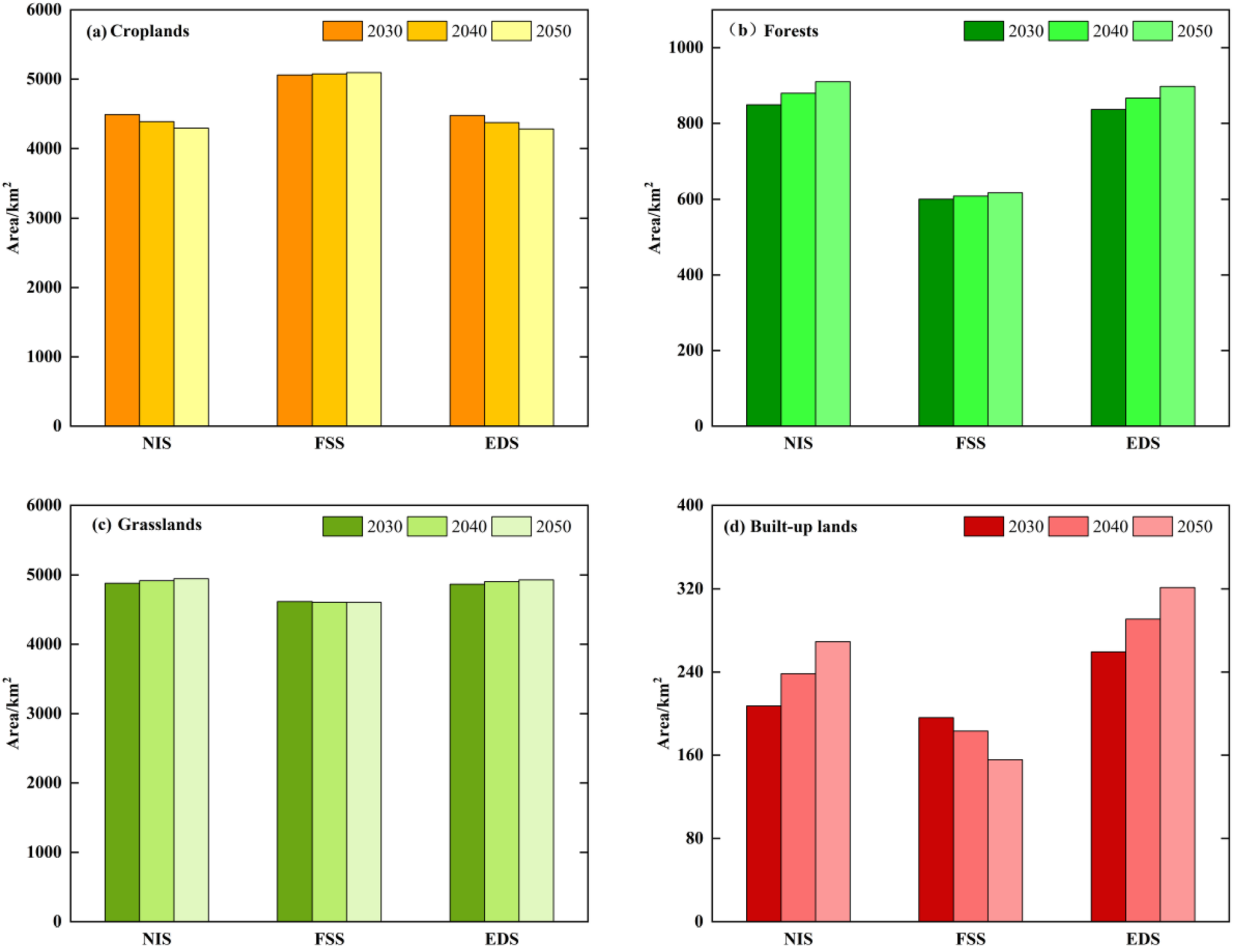
Landuse area changes for croplands, forests, grasslands, and built-up areas under three scenarios (2030–2050).

**Fig. 8:**
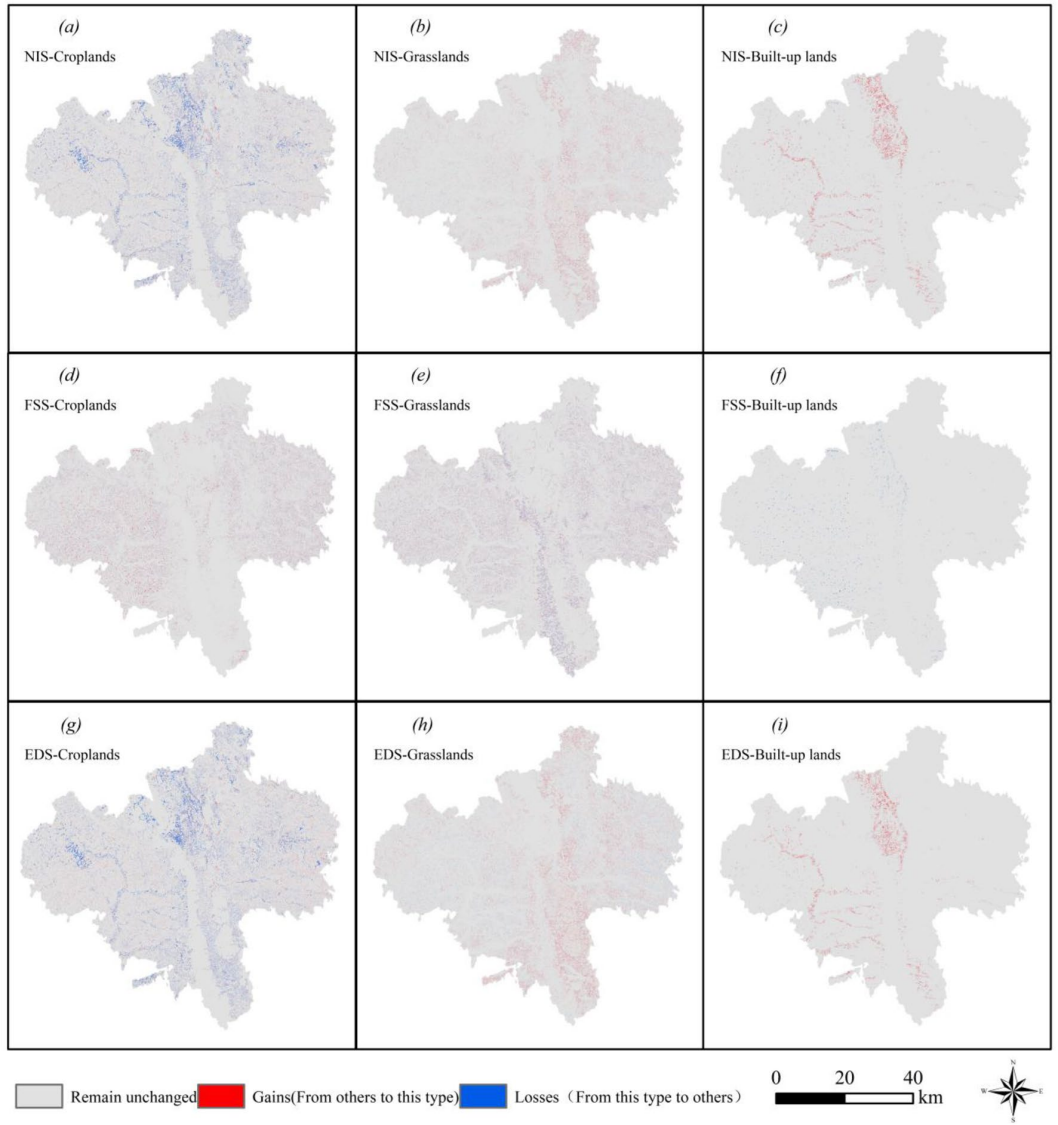
Spatial changes in croplands, grasslands, and built-up areas under the NIS/FSS/EDS (2030–2050). Created with ArcGIS 10.8.

The spatial patterns remained consistent across scenarios (Fig. 6). Croplands and grasslands dominated in northern, northwestern, and northeastern Guyuan (Xiji, Yuanzhou, and Pengyang counties) and showed fragmented distributions. Forests formed linear patterns in the central–southern areas (Pengyang and Jingyuan counties), while built-up lands clustered in the northern and southeastern zones (Yuanzhou and Jingyuan counties) as scattered and contiguous patches. Quantitative analysis (Fig. 7) revealed distinct trends: Under the NIS, croplands decreased from 4,489.64 km² (2030) to 4,294.09 km² (2050) compared with the 2020 baseline of 4,602.4 km². The EDS showed a 319.05 km² loss of cropland, whereas under the FSS, cropland areas increased by 491.49 km² (5,093.89 km² total). Spatial expansions under the FSS were concentrated in southern–central Xiji and southeastern Pengyang (Fig. 8). NIS-driven cropland losses occurred in central Yuanzhou and southern Jingyuan, with sporadic expansion risks in western Guyuan.

Under the FSS (2030–2050), grassland and forest areas remained stable with minimal fluctuations. Compared to the 2020 baselines (grasslands: 4,814.43 km²; forests: 774.06 km²), the NIS showed modest increases for both land types (Fig. 7b and c, respectively). The FSS exhibited significant forest loss (−156.95 km²) and slight grassland reduction. NIS-driven grassland expansion (Fig. 8b) mainly occurred in the mid- to high-elevation zones of Longde County and Jingyuan County. The ESS exhibited comparable grassland expansion to that under the NIS, except for sporadic decreases in western Guyuan. The FSS showed distinct patterns, with southern decreases and localized fluctuations elsewhere.

Built-up areas expanded at 1.26 km²/year (NIS) and 2.99 km²/year (EDS) from the baseline of 231.46 km² in 2020 (Fig. 7d). Urban growth was clustered contiguously in Yuanzhou under both the NIS and EDS, forming linear patterns in Longde and Xiji. The FSS showed the opposite trend, as the net urban area decreased.

### Water supply–demand dynamics

The Ningxia Water Resources Bulletin provides Guyuan’s 2020 water resources at 5.97 × 10⁸ m³. Calibration revealed Z = 11 as the optimal precipitation parameter, yielding 6.14 × 10⁸ m³ of simulated supply matching the observed data.

The data (Table 8) indicated a worsening supply–demand imbalance. Water supply fluctuated mildly from 7.54 × 10⁸ m³ (2030) to 6.95 × 10⁸ m³ (2050) (−7.8% over 20 years), indicating limited landuse impacts. However, the water demand increased by 43.3% from 15.44 × 10⁸ m³ to 22.13 × 10⁸ m³ from 2030 to 2050, indicating that landuse changes exert a greater influence on the demand than on the supply, thus driving structural imbalances.

**Table 8:** Water supply–demand dynamics under different scenarios (2030–2050).

Year	Water yield supply (×10^8^ m³)			Water yield demand (×10^8^ m³)		
	NIS	FSS	EDS	NIS	FSS	EDS
2030	7.54	7.48	7.52	15.44	16.87	15.39
2040	7.58	7.46	7.56	18.58	21.07	18.46
2050	6.95	6.76	6.92	22.13	25.78	22.01

**Fig. 9:**
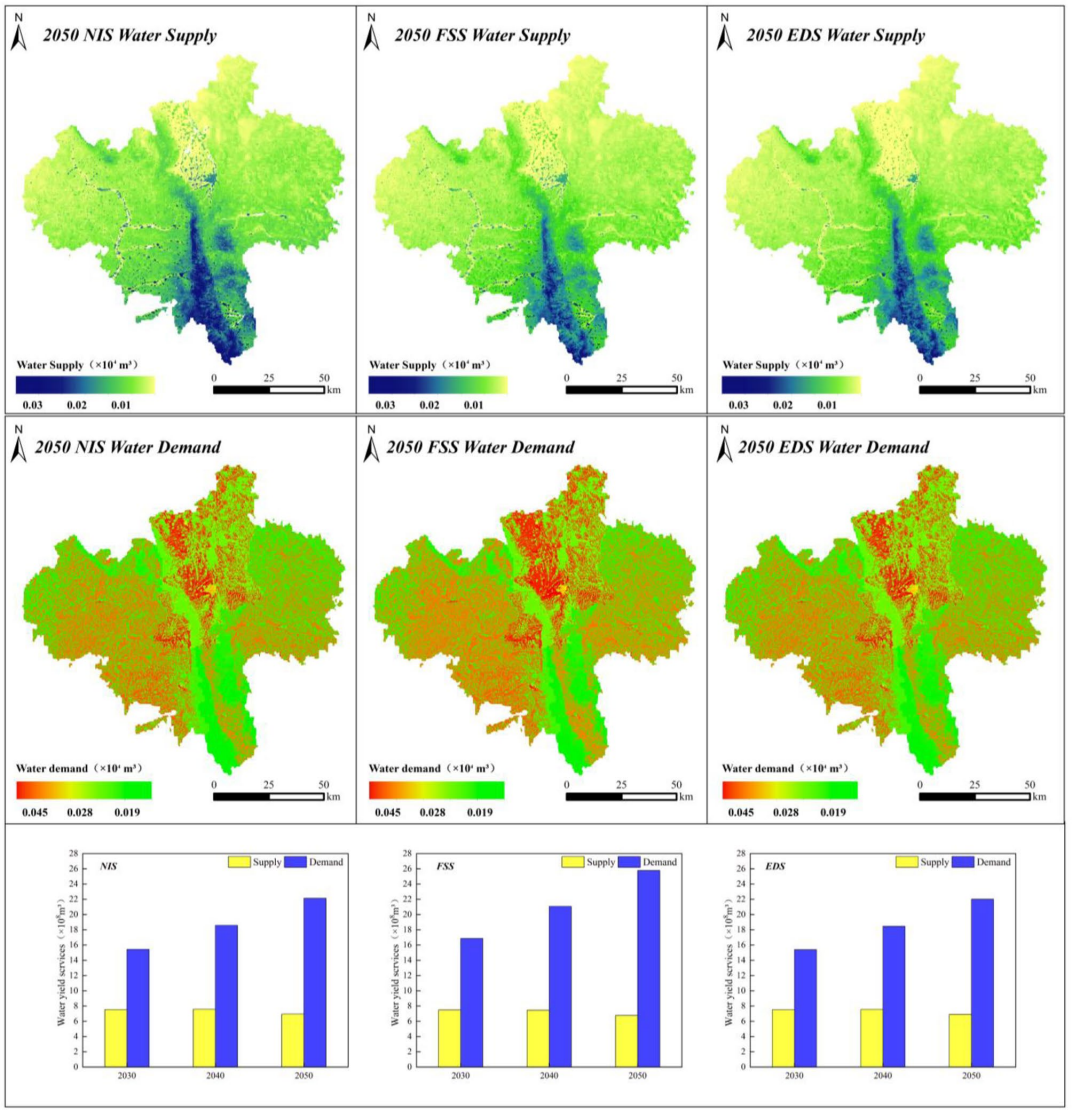
Geographical dispersion of the water supply–demand across the various scenarios in 2050, with temporal changes from 2030–2050. The maps were created with ArcGIS 10.8.

**Fig. 10:**
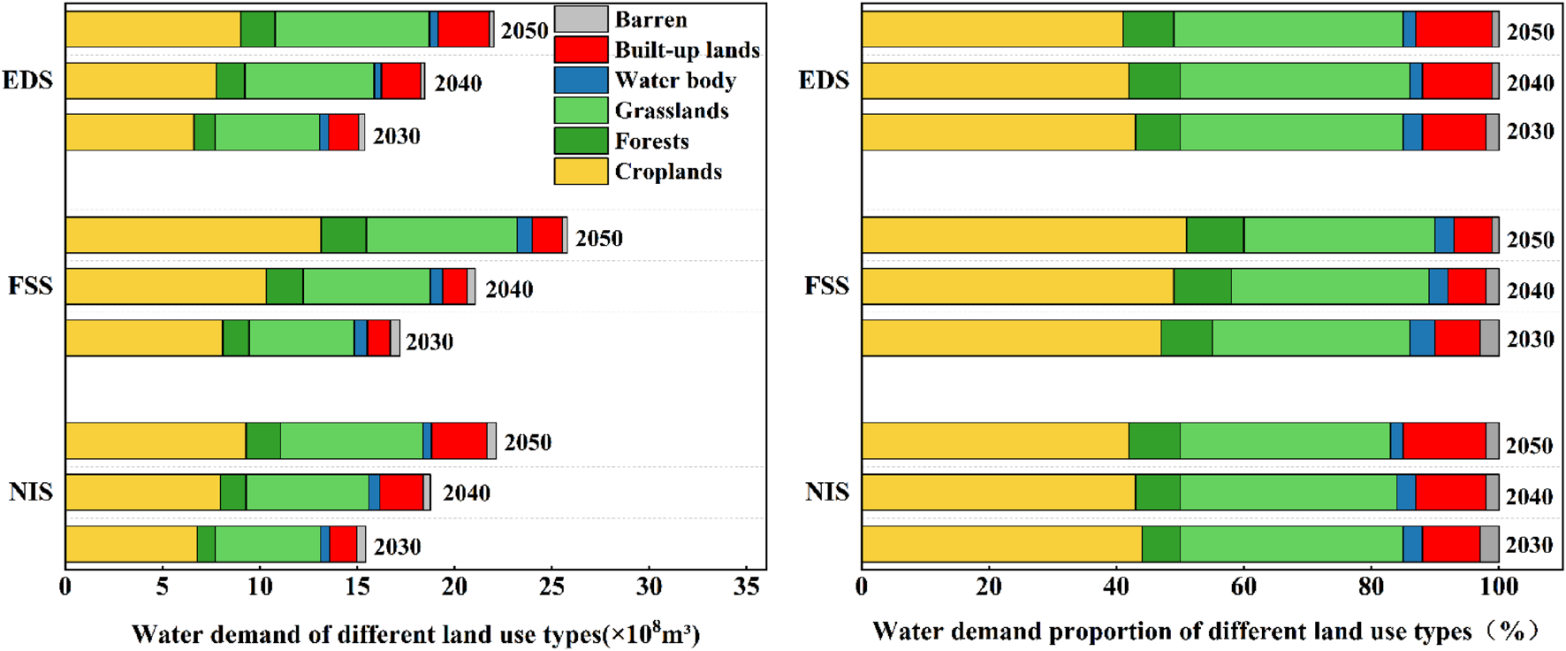
Water demand by landuse type (left) and demand composition (right) under the NIS/FSS/EDS (2030–2050).

Spatially heterogeneous landuse drives notable water resource supply–demand imbalances across the region, resulting in distinct spatial zoning (Fig. 9). Supply-side analysis revealed water provisioning capacity deficits constrained by forest/unused land distributions, with critical shortages concentrated in ecologically fragile zones, such as eastern Longde, southern Jingyuan, and northern Xiji counties. Demand-side analysis revealed a high-water-intensity core encompassing northwestern cropland clusters and southwestern urban expansion zones in Guyuan, with spatial polarization intrinsically linked to regional landuse restructuring. As shown in Fig. 10, arable land always occupies a dominant position in the water demand structure, and the proportion of its water demand under the FSS continues to increase and exceeds 51% from 2030 to 2050. Comparative analysis revealed that compared with the decreasing trend in the water supply capacity under the NIS and EDS, the increase in the water demand triggered by the expansion of developed land was similar across the scenarios. The water demand from developed land increased by 44% from 0.09 × 108 m³ to 0.13 × 108 m³, and the urbanization process exerted pressure on the regional water resource system. Space–time dynamics of resource allocation imbalances critically inform territorial spatial governance.

**Fig. 11:**
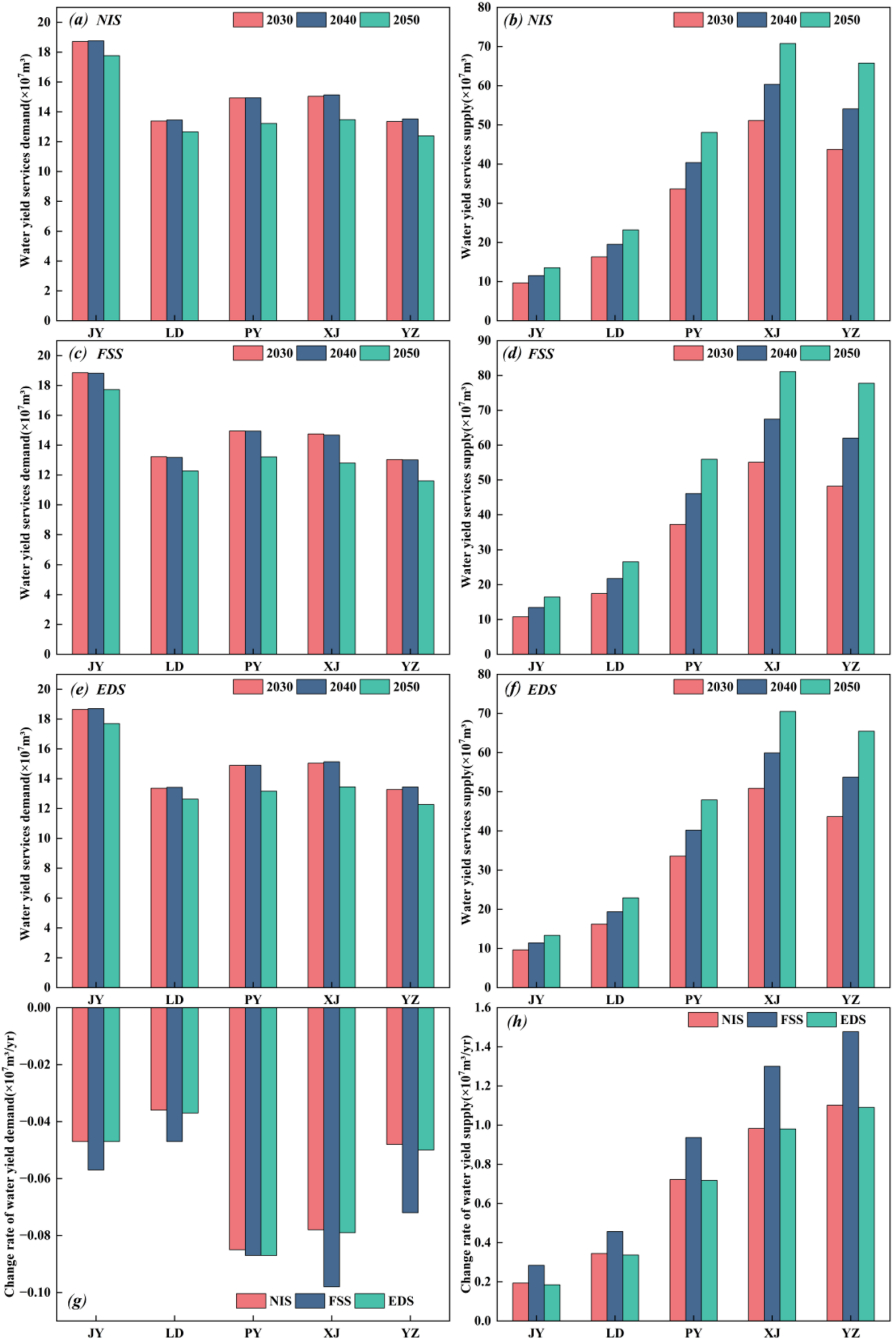
County-scale water budgets: 2030–2050 scenario-based supply (left) vs. demand (right) in Guyuan. JY, LD, PY, XJ and YZ in the subfigure denote Jingyuan, Longde, Pengyang, Xiji and Yuanzhou (districts and counties), respectively.

A water resource analysis at the county scale revealed (Fig. 11) that Yuanzhou District was characterized by the lowest regional water production capacity, whereas Pengyang County experienced the largest water resource decrease under the EDS, with an inter-annual decrease of 830,000 m^3^ in water production. From 2030 to 2050, all administrative units in the region showed a continuous decrease in water production. In contrast, the scale of the water demand in the counties under the various scenarios significantly increased. The counties of Yuanzhou and Xiji performed well in terms of the incremental water demand (average annual change) and the total scale, with the former reaching the regional extreme. The total water demand in the two areas was high, which reflects the gravity of the future conflict between water resource supply and demand in the region.

### Evolution of water security risk

**Fig. 12:**
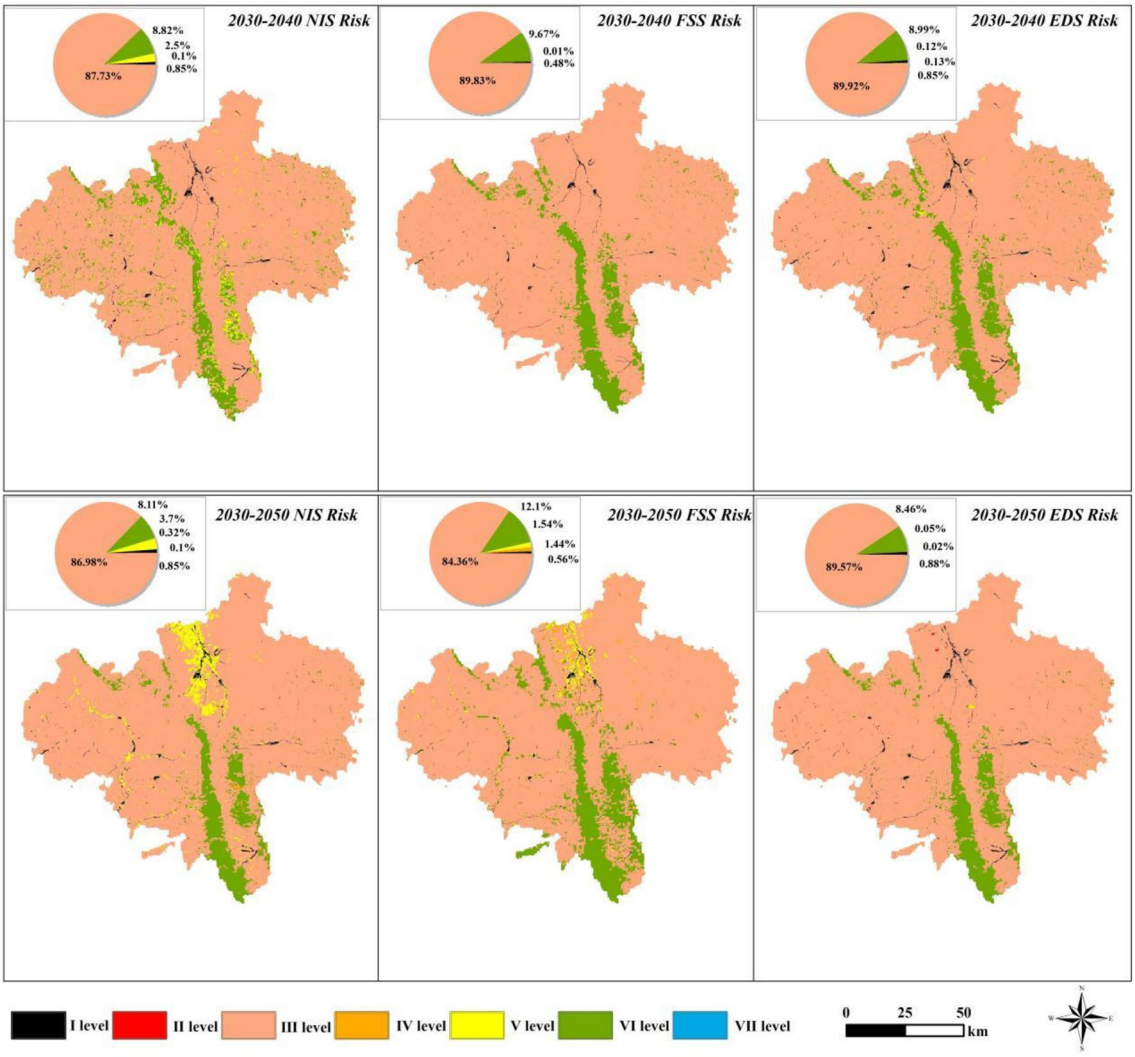
Water security risks under the NIS/FSS/EDS scenarios from 2030–2040 and 2030–2050. Created with ArcGIS 10.8.

An analysis of the results shown in Fig. 12 revealed that the water supply–demand risk exhibits notable spatiotemporal heterogeneity between 2030 and 2050. By 2030, approximately 90% of the city’s area will experience a Grade III (endangered) water supply risk; however, the risk level shows a decreasing trend over time. By 2050, the proportion of the Grade III risk area under the FSS decreased by 5.53% to less than 85%. Areas at a Grade II (critically endangered) risk show differential expansion. Under the NIS, the proportion of this risk zone increased from 0.01% in 2030 to 0.02% in 2050 and was mainly distributed in the northern grassland (58.72%) and woodland (35.38%). The expansion area under the EDS was dominated by developed land (60.23%), followed by grassland (20.58%) and woodland (13.27%). Notably, there was spatial similarity in the pattern of Grade II risk expansion under the two scenarios. In Grade IV (hazardous) risk areas, all scenarios showed an increasing trend, specifically a 1.44% increase under the FSS and a 1.2% rise under the NIS, and their spatial evolution paths were characterized by the three-stage feature of central agglomeration–axial diffusion–piecewise spreading. The evolution trajectory of the FSS was particularly significant. In 2030, water supply gaps began to appear in the central part of Yuanzhou District and in local arable land in Jingyuan County; by 2050, the risk areas had expanded to most of Yuanzhou District, the main area of Jingyuan County, and sporadic plots in Pengyang County, which could lead to the formation of contiguous risk zones.

Time series comparison analysis showed that the developed land under each scenario continued to be at high risk from 2030 to 2040. Based on landuse types, the spatial pattern of secondary risk zones exhibits a significant land type orientation, with ecological land dominating under the NIS and developed land dominating under the EDS. This variation reflects different pressures on the water resource system in different development models, with the NIS affecting mainly natural ecosystems and the EDS exerting more pressure on built-up land.

### Approaches to reducing water allocation issues in Guyuan

By integrating the analysis of water stress dynamics and use pattern evolution across multiple scenarios from 2030 to 2050 for Guyuan city, we formulated mitigation strategies to optimize water allocation among the agricultural, ecological, urbanization, and industrial sectors. Within agriculture, systemic fragility and imbalances stem from crop yield overemphasis and extensive management practices. According to the Ningxia Statistical Yearbook, traditional flood irrigation methods remain dominant in Guyuan city, accounting for more than 65% of agricultural irrigation. This results in a water resource utilization rate lower than 40%, which is significantly lower than the value of 85% for drip irrigation and the value of 75% for sprinkler irrigation. Field seepage and evaporation losses account for 52% of the total water use^44^, exacerbating the water resource supply–demand imbalance. Due to the characteristics of loess hilly arid regions, Guyuan city should promote the use of flexible water tanks and rainwater harvesting technologies. These efforts can increase annual water storage by 0.12 × 10^8^ m³^45^, effectively alleviating water resource constraints.

Ecologically, the water utilization discrepancies between anthropogenic activities and ecosystems are increasing^46^, particularly in the fragile loess hilly arid region. To meet the ecological governance needs of Guyuan city, in vulnerable areas, such as Yuanzhou District, Pengyang County, and Xiji County, the rational allocation of afforested land and grassland proportions should be achieved by constructing stepped forest-grass composite systems to increase soil and water conservation efficiency levels. Additionally, through heterogeneous patch design and native species combinations, the landscape diversity and ecological stability of forest-grass communities can be enhanced^47^, thereby reducing system water stress risks.

Urban–industrial expansion in Guyuan city substantially increases water consumption and pollution, acutely intensifying hydrological stress, particularly in Yuanzhou District and Xiji County. Advancing water-saving technologies and reclaimed water reuse in industries can optimize the enterprise-level water efficiency^48^. Additionally, enhancing public conservation awareness and rigorous pollution control can mitigate water risks while supporting carbon neutrality objectives.

## Discussion

### Analysis of future landuse changes across different scenarios

In this study, we used a PLUS model to simulate and analyze the evolution of landuse in Guyuan city from 2020 to 2050 across multiple scenarios. The simulations revealed that the transformations of farmland and ecological land show significant spatial heterogeneity. Under the NIS, the forest area increased from 848.88 km² in 2030 to 909.89 km² in 2050, an increase of 7.12%, and the grassland area increased from 4828.85 km² to 4985.75 km², an increase of 3.25%. The expansion hotspots were mainly located in the high-elevation regions of southern Jingyuan County and Pengyang County (Figs. 6 and 7, respectively). The loss of farmland was characterized by terrain selection. Under the NIS, the farmland area decreased by 195.55 km², a reduction of 4.36%, with 83.2% of the converted farmland distributed in hilly areas with slopes greater than 15°. The spatial transition matrix indicated that 76.4% of the lost farmland was transformed into forests and grassland. Research has indicated that while large-scale farmland-to-forest programs can enhance ecological benefits, they may impose a crowding-out effect on high-quality farmland with a slope of less than 6°, leading to a decrease in the grain yield per unit area^49^. Sustainable development can be achieved through the coordinated delineation of red lines to protect farmlands and ecosystems.

Under the FSS, regional farmland significantly expanded. The newly cultivated areas were mostly concentrated in regions with low natural endowments, such as Xiji County and Longde County, which are generally characterized by high altitude, low soil fertility, and high evapotranspiration. To address the environmental concerns, local governments should implement land consolidation and engineering improvement measures to increase farming suitability^50^. However, these measures could directly increase the marginal cost of agricultural production. Additionally, large-scale farmland expansion could lead to significant encroachment on forest and grassland in the southern part of Guyuan city (Fig. 8). This drastic transformation of landuse types may not only pose a threat to the integrity of regional ecosystems but also negatively impact surface vegetation cover and soil structure stability^51,52^, thereby posing potential risks to regional soil and water conservation functions.

Across the EDS framework, the continuous expansion of developed land caused large-scale encroachment and degradation of farmland, forestland, and grassland. This process poses a direct threat to regional agricultural production foundations and ecosystems and exacerbates the tension of human-land relationships in the region by compressing the carrying space of natural resources.

The comprehensive scenario simulation results demonstrated that the EDS has notable drawbacks. The disorderly expansion of developed land poses a threat to food security and increases ecological degradation risks, creating dual pressures for development and protection. Relying solely on the FSS or NIS also fails to achieve system optimization, as the former leads to ecological service function imbalance due to excessive cultivation, whereas the latter results in inefficient resource allocation due to neglected development needs. Future management strategies should overcome the limitations of single scenarios by establishing a multi-objective coordination mechanism for agricultural production, ecological protection, and urban - rural development. A dynamic balance in landuse structure adjustment, efficient water resource allocation, and ecological policy innovation should be achieved. This adaptive strategy, in which development quality and ecological safety are balanced, may become a key approach to resolving the coordination challenges of Guyuan city’s human - land coupling system.

### Assessing the risks of the water supply and demand services

The water resource supply–demand risks in Guyuan city exhibit significant systemic characteristics. As regional development progresses, contradictions between limited water resource carrying capacity and diversified water demands gradually emerge^6,53^. Local governments face multiple constraints in coordinating the allocation of water for production, living, and ecological purposes. Ecological processes driven by different landuse patterns significantly influence regional water provisioning capacity. Therefore, in this study, we employed multi-scenario landuse simulations to analyze the mechanisms through which spatial pattern evolution impacts supply–demand risks. The simulation results, shown in Fig. 12, indicate that over the next 30 years, most areas of the city will experience endangered water resource supply–demand risks, with agricultural water deficits persisting across various scenarios.

Current water resource development and use data revealed structural contradictions. The overreliance of agricultural irrigation methods on groundwater extraction has caused a continuous decline in the groundwater level in Xiji County. In Pengyang County, on the southern slope of Mount Liupan, due to the steep terrain and thin aquifers, coupled with the agricultural water demand, there is a risk of deep groundwater overexploitation, which may cause irreversible damage to the regional ecosystem^54^. The FSS showed that from 2030 to 2050, the arable land area will significantly increase (Fig. 7). However, the newly added arable land is concentrated in water-stressed regions^55,56^. The significant spatial distribution differences between these areas and the water supply capacity (Fig. 10) may exacerbate supply–demand conflicts and the potential conflicts between agricultural production and ecological security^57^. Against the backdrop of global warming, the increasing frequency of droughts^58^will amplify regional supply–demand risks through enhanced evaporation and precipitation fluctuations, which pose a threat to the stability of agricultural systems.

EDS analysis revealed amplified risk propagation from developed land expansion. From 2030 to 2050, the land demand in areas with dense industrialization and urbanization continued to increase (Fig. 8). The expansion of developed land resulted in the direct encroachment onto surrounding high-quality farmland and forestland. This not only reduces the natural system’s water retention capacity but also exacerbates water supply–demand risks through the compression of ecological space^59^. The interplay between landuse change and the water resource system highlights the complexity of resource allocation in regional development and the vulnerability of ecological security.

In terms of the water supply, the region’s precipitation is unevenly distributed in time and space, with high evaporation intensity. When combined with the unique topography of the loess hilly arid region, which results in low rainfall interception capacity and low groundwater recharge efficiency levels, these natural constraints form the fundamental conditions for resource-type water scarcity^60^. In terms of the water demand, in addition to the aforementioned factors, desertification control and vegetation restoration projects in Guyuan city exhibit significant consumption of ecological water, leading to a gradual increase in water resource supply–demand risks.

### Impact and recommendations for dryland agriculture development

As a typical agricultural region in a semiarid loess hilly area of northwest China, Guyuan city faces the dual challenges of rigid water resource constraints and ecological fragility. The ongoing intensification of supply–demand risks render water the core limiting factor for sustainable agricultural development^61^. Additionally, frequent droughts and severe soil erosion lead to nutrient loss in the plow layer, directly reducing farmland quality and productivity^62^. Therefore, upgrading agricultural infrastructure and applying water-saving technologies are crucial for regional development. The large-scale promotion of drip irrigation technology is crucial for reducing agricultural water supply–demand risks^63^. Chinese agricultural production is still dominated by smallholder farming, and their farming habits and technology adoption preferences significantly influence the choice of sustainable agricultural strategies and the maintenance of regional ecological security^64^. Consequently, Guyuan city should actively respond to national policies by implementing the construction of high-standard farmland, thereby enhancing agricultural productivity and water-saving capacity. However, there is an insurmountable technological gap between smallholder farming and large-scale enterprise-led high-standard farmland development. Empowering farmers with advanced agrotechnologies (e.g., conservation tillage, drip irrigation, and remote sensing monitoring) for soil structure optimization and enhanced hydro-ecological conservation is imperative^65^.

Balancing water use for domestic, agricultural, and industrial purposes in Guyuan city is crucial for alleviating water scarcity, promoting regional agricultural development, and improving the ecological environment. First, reforms in water resource efficiency should be implemented, along with precision water use technologies. Adopting shallow subsurface drip irrigation combined with soil moisture monitoring to establish smart irrigation zones can reduce deep percolation losses during irrigation^66^. Second, optimizing crop structures by promoting the use of intercropping models, such as potato–Korshinsk peashrub, can increase the efficiency of precipitation use^67^. Third, a linkage mechanism integrating agricultural water conservation and ecological compensation should be established. Government can reward farmers for adopting water-saving initiatives to increase their enthusiasm^68^. Finally, water resource allocation should be balanced and quantified between desertification control and vegetation restoration projects, agricultural production and ecological protection^19,69^.

### Applicability and limitations of the study

In this study, we constructed multiple future landuse scenarios based on the PLUS model and evaluated water resource supply and demand conditions, offering a valuable perspective for understanding the evolution of human-land relationships in semiarid regions. From a methodological perspective, the PLUS model captures landuse changes by integrating natural and socioeconomic driving factors, with a relatively flexible configuration of landuse transition rules. The water resource assessment module of the InVEST model is grounded in the quantitative analysis of factors such as precipitation, vegetation, and soil. The underlying logic of water production and consumption processes may be broadly applicable to most regions where the water resource supply–demand chain is clearly defined. Therefore, for arid and semiarid regions experiencing dynamic landuse transformations and water resource supply–demand pressures, the core methodologies of this framework can serve as a reference. With appropriate parameter calibrations and detailed adjustments tailored to the natural and socioeconomic conditions of the target region, meaningful insights can be derived.

Nevertheless, this study is subject to certain limitations. The current simulation relies primarily on existing socioeconomic and climatic conditions but does not fully incorporate the potential impacts of future climate change scenarios and socioeconomic development pathways on landuse. This may affect the model ability to accurately represent dynamic feedback mechanisms in long-term land system evolution, particularly in climate-sensitive areas. Furthermore, the projection of industrial and domestic water demand does not adequately account for variations in the industrial water use efficiency across different sectors or their temporal dynamics. This limitation is especially pronounced in rapidly industrializing regions. Moreover, policy interventions such as ecological conservation and agricultural restructuring are only indirectly incorporated through binding constraints, necessitating development of more adaptive quantitative modeling pathways. Additionally, the water resource assessment focuses on point-source supply and terminal demand, while systematic incorporation of ecosystem service flows remains absent—potentially compromising the analytical depth regarding cross-regional water dependencies and system resilience.

## Conclusions

As a typical semiarid loess hilly region, Guyuan city faces three potential development scenarios: NIS, FSS and EDS. In this study, we projected landuse patterns in Guyuan city from 2030 to 2050 and analyzed water supply–demand situations and risks under each scenario. The results indicated that while landuse exerts negligible influence on the water yield, it significantly increases the water demand by 43.3%, with 90% of Guyuan city’s area expected to experience water supply–demand risk from 2030 to 2050. Under the NIS, severe risks concentrated in grassland and forestland were obtained. Under the EDS, risks extended to grassland, forestland, and developed land. These findings underscore the heightened vulnerability of dryland ecosystems and socioeconomic systems to hydro–climatic stress. On the basis of these findings, various mitigation strategies have been proposed, including developing water-saving agriculture, adjusting crop structures, improving industrial water-saving technologies, and increasing the water use efficiency, all of which aim to increase agricultural sustainability.

## Data Availability

Data is provided within the manuscript.
